# Creosote Versus Carbolic Acid

**Published:** 1888-06

**Authors:** 


### CREOSOTE VERSUS CARBOLIC ACID.

There is a curious lack of comprehension of the wide difference between creosote and carbolic acid. Some of our most intelligent men use the terms interchangeably and as synonyms. The fact is, the two articles have little in common. As medicinal agents they are, or should be, employed for widely different purposes. And yet, dentists frequently recommend the one when they mean the other. Creosote has but a very unimportant place in the dental pharmacopœia, while carbolic acid is, perhaps, employed more frequently than any other remedy. There are very few of the dentists who so commonly advise its use in their writings or speeches, who even have it in their cases. Indeed, it is not fit for exhibition in the operating room, because of its oppressively vile and penetrating odor. Let us review some of the characteristics of each, that it may be judged which of the two is best adapted to dental wants.

Creosote is obtained from wood, carbolic acid from coal tar.

Creosote, when pure, is a liquid, carbolic acid a solid.

Creosote will not coagulate collodion, carbolic acid will.

Creosote will not produce a blue color by reaction with muriatic acid, carbolic acid will.

Creosote forms solutions with eighty parts of water, and with one-tenth of one part, carbolic acid with twenty parts.

Creosote is not a cauterant, carbolic acid is.

Creosote is a narcotic, carbolic acid is an irritant.

Creosote is an oil, carbolic acid is a phenylic alcohol.

Creosote has for its formula $C_8 H_{10} O_2$, carbolic acid is composed of $C_6 H_5 HO$.

Creosote is a soothing application to ulcers and for putrid sore throat, carbolic acid is exceedingly irritating.

Creosote is styptic and astringent, carbolic acid is not.

Finally, and most important, creosote is not a germicide or a disinfectant at all, while carbolic acid is one of the most powerful with which we are acquainted.

Will not dentists take note of these differences, and use and recommend the two drugs intelligently. Medical men who know the characteristics of each, are not impressed with our chemical and pharmaceutical lore when they hear us prescribing creosote for the septic canal of a tooth.

